# Motor cortical function and the precision grip

**DOI:** 10.14814/phy2.12120

**Published:** 2014-12-11

**Authors:** Nimeshan Geevasinga, Parvathi Menon, Matthew C. Kiernan, Steve Vucic

**Affiliations:** 1 Sydney Medical School Westmead, University of Sydney, Sydney, NSW, Australia; 2 The Brain and Mind Research Institute, University of Sydney, Sydney, NSW, Australia

**Keywords:** Cortical excitability, power grip, precision grip

## Abstract

While task‐dependent changes in motor cortical outputs have been previously reported, the issue of whether such changes are specific for complex hand tasks remains unresolved. The aim of the present study was to determine whether cortical inhibitory tone and cortical output were greater during precision grip and power grip. Motor cortex excitability was undertaken by using the transcranial magnetic stimulation threshold tracking technique in 15 healthy subjects. The motor‐evoked potential (MEP) responses were recorded over the abductor pollicis brevis (APB), with the hand in the following positions: (1) rest, (2) precision grip and (3) power grip. The MEP amplitude (MEP amplitude _REST_ 23.6 ± 3.3%; MEP amplitude _PRECISION_ _GRIP_ 35.2 ± 5.6%; MEP amplitude _POWER_ _GRIP_ 19.6 ± 3.4%, *F* = 2.4, *P* < 0.001) and stimulus‐response gradient (SLOPE_REST_ 0.06 ± 0.01; SLOPE_PRCISION_ _GRIP_ 0.15 ± 0.04; SLOPE _POWER_ _GRIP_ 0.07 ± 0.01, *P* < 0.05) were significantly increased during precision grip. Short interval intracortical inhibition (SICI) was significantly reduced during the precision grip (SICI _REST_ 15.0 ± 2.3%; SICI _PRECISION_ _GRIP_ 9.7 ± 1.5%, SICI _POWER_ _GRIP_ 15.9 ± 2.7%, *F* = 2.6, *P* < 0.05). The present study suggests that changes in motor cortex excitability are specific for precision grip, with functional coupling of descending corticospinal pathways controlling thumb and finger movements potentially forming the basis of these cortical changes.

## Abstract (precis)

This manuscript establishes that specific cortical mechanisms underlie the maintenance of the precision grip. The mechanisms appear distinct to the processes maintaining the power grip.

## Introduction

Precision grip is defined as the act of grasping an object between the opposed tips of the thumb and index finger, and is vital for performance of skilled hand movements required for everyday function (Lemon and Griffiths 2005; Lemon 2010). In humans, the execution of the precision grip is dependent on the ability to perform fine fractionated finger movements (Lemon et al. 1996; Lemon 1997, 2010; Lemon and Griffiths 2005), largely mediated by a co‐ordinated activity of the thenar group of muscles, including the abductor pollicis brevis (APB), together with first dorsal interosseous (FDI) (Napier 1956; Long et al. 1970; Jeannerod 1986; Forssberg et al. 1991; Maier and Hepp‐Reymond 1995; Marzke 1997; Marzke et al. 1998; Johanson et al. 2001; Brochier et al. 2004).

The neural processes mediating the execution of a precision grip remain to be fully elucidated, although neurophysiological studies in animals and humans have identified the importance of motor cortical neural networks, particularly the corticomotoneuronal component which projects directly onto spinal motor neurons (Lemon and Griffiths 2005). Increased corticomotoneuronal activity has been reported in monkeys during precision grip, and postulated to represent an adaptive cortical response required for execution of fractioned finger movements (Muir and Lemon 1983; Buys et al. 1986; Lemon et al. 1996; Lemon 1997, 2008, 2010).

Lesion studies in nonhuman primates involving complete or partial interruption of the corticospinal tract (CST) resulted in permanent deficits in skilled hand movements, particularly fine fractionated finger movements (Lawrence and Kuypers 1968; Sasaki et al. 2004; Freund et al. 2006; Courtine et al. 2007; Lemon 2008). Underscoring the importance of motor cortical processes in the precision grip is the finding of marked species variation in the degree of corticomotoneuronal system development, such that the corticomotoneuronal system is most developed in humans with the highest index of hand dexterity, and least developed in rodents which exhibit a low index of dexterity (Lemon and Griffiths 2005; Lemon 2008).

Transcranial magnetic stimulation (TMS) techniques have provided a unique opportunity to noninvasively assess the excitability properties of motor cortical networks and the corticomotoneuronal system in humans (Kujirai et al. 1993; Nakamura et al. 1997; Hanajima et al. 1998; Vucic et al. 2006; Chen et al. 2008). The importance of motor cortical networks in regulating hand function is underscored by differences in the potency of intracortical inhibitory and facilitatory directed toward the distal and proximal upper limb muscles (Abbruzzese et al. 1999). Short interval intracortical inhibition (SICI), a biomarker of inhibitory cortical networks (Ziemann 2003), appears to be significantly greater when recorded over thenar muscles compared to the biceps brachii (Abbruzzese et al. 1999), thereby suggesting a greater potency of inhibitory cortical networks directed to the thenar muscles, in keeping with the importance of thenar muscles in the execution of the precision grip (Abbruzzese et al. 1999; Eisen and Kuwabara 2012).

Underscoring the importance of cortical processes in the regulation of hand function are findings of task‐dependent changes in motor cortical network excitability (Flament et al. 1993; Huesler et al. 1998; Hasegawa et al. 2001; Devanne et al. 2002; Stinear and Byblow 2004; Kouchtir‐Devanne et al. 2012). Specifically, an increase in MEP amplitude has been previously documented during the execution of the precision grip, which was independent of background electromyography activity (Flament et al. 1993; Schieppati et al. 1996; Huesler et al. 1998; Kouchtir‐Devanne et al. 2012). Task‐dependent disinhibition of the motor cortex, as indicated by reduction in short and long interval intracortical inhibition, has also been reported, thereby suggesting that the increase in MEP amplitude may be mediated by disinhibition of motor cortical networks (Kouchtir‐Devanne et al. 2012). Cortical inhibition, however, was measured at one time point (Kouchtir‐Devanne et al. 2012), thereby potentially providing an incomplete insight into the cortical networks mediating precision grip, especially in light of the fact that short interval intracortical inhibition is comprised of physiologically distinct phases (Fisher et al. 2002; Vucic et al. 2006, 2009, 2011). In addition, most studies recorded responses from the first dorsal interosseous (FDI) muscle, thereby precluding conclusions about the cortical processes subserving the thenar group muscles. Confirmation of task‐dependent changes in cortical excitability from thenar muscles, which were similar to those recorded over FDI, may lend further support to the notion that functional coupling of descending corticomotoneuronal processes may underlie the precision grip. Consequently, the present study utilized threshold tracking TMS techniques to further delineate motor cortical processes mediating the precision grip in humans, particularly to determine whether task‐dependent changes in motor cortex excitability were evident when recording from the thenar muscles, and whether cortical disinhibition was mediated by the physiologically distinct networks.

## Materials and Methods

### Subjects

Studies were undertaken on 15 right‐handed healthy volunteers (six men, nine women; mean age 36 years, age range 22–53 years). None of the subjects had symptoms or clinical signs of central or peripheral nervous system dysfunction, and were not receiving psychotropic medications at the time of testing. Subjects gave written informed consent to the procedures, and all procedures were approved by the Western Sydney Local Health District Human Research Ethics Committee.

### Experimental tasks

#### Transcranial magnetic stimulation

Transcranial magnetic stimulation studies were undertaken by applying a 90 mm circular coil connected to two high‐power magnetic stimulators connected via a BiStim device (Magstim Co., Whitlands, South West Wales, UK). The coil position was adjusted such that an optimal stimulating site was determined as indicated by a point on the vertex at which a maximal motor‐evoked potential (MEP) amplitude was evoked by the smallest TMS current. The MEP response was recorded over the abductor pollicis brevis (APB) muscle. The circular coil was chosen over a focal (figure‐of‐eight) coil as the former was easier to use with less frequent overheating of the coil itself. Importantly, no qualitative differences in the pattern of inhibition and facilitation have been reported between the circular coil and focal (figure‐of‐eight) coils (Abbruzzese et al. 1999).

Given that the aim of this study was to compare the effects of precision grip on cortical excitability, TMS studies were undertaken with the hand positioned in three different postures. First, the subjects were instructed to supinate the forearm, such that the palm was facing upwards with the thumb relaxed, termed the “*neutral*” position. Subsequently, the subjects were instructed to hold a pen (10 cm in length and 5 g in weight) in the dominant hand, between thumb and index finger, executing a *precision grip*. Lastly, the subjects were instructed to grip the same pen in a *power grip*, with the pen grasped by the whole hand as described previously (Flament et al. 1993). In order to avoid dynamic influences of arm posture, the elbow was semiflexed, forearm semipronated and the wrist was maintained in a neutral position, preventing volarflexion or dorsiflexion of the wrist for the duration of the experiment, by strapping the forearm to the chair handle. Auditory and visual electromyography (EMG) feedback was provided from the APB muscle to ensure that EMG activity was ~10% of maximal voluntary contraction for all three positions. Short interval intracortical inhibition, intracortical facilitation, resting motor threshold, MEP amplitude, MEP latency and central motor conduction time were measures during each task according to methods described below.

Short interval intracortical inhibition and ICF were assessed by utilizing the paired‐pulse threshold tracking TMS according to a previously reported technique (Fisher et al. 2002; Vucic et al. 2006). Briefly, the MEP amplitude was fixed and changes in the test stimulus intensity required to generate a target response of 0.2 mV (±20%), when preceded by sub‐threshold conditioning stimuli, were measured. Motor threshold (MT) was defined as the stimulus intensity required to maintain the target MEP response of 0.2 mV (±20%). A value of 0.2 mV was selected as the tracking target, rather than the conventional value of 0.05 mV used in the constant stimulus TMS technique (Chen et al. 2008), given that the former target response (0.2 mV) lies in the middle of the linear logarithmic stimulus‐response relationship over a hundred‐fold range of responses from about 0.02 to 2 mV (Fisher et al. 2002). As such, larger variations in the MEP amplitude translated to smaller variations in the stimulus intensity (the outcome variable), potentially enabling more accurate recordings of TMS parameters.

Short‐interval intracortical inhibition (SICI) was determined over the following interstimulus intervals (ISIs): 1, 1.5, 2, 2.5, 3, 3.5, 4, 5, and 7 ms, while intracortical facilitation (ICF) was measured at ISIs of 10, 15, 20, 25 and 30 ms. Stimuli were delivered sequentially as a series of three channels: channel 1: stimulus intensity, or threshold (% maximal stimulator output) required to produce the unconditioned test response (i.e., MT); channel 2: subthreshold conditioning stimulus (70% MT); and channel 3 tracks the stimulus (% maximal stimulator output) required to produce the target MEP when conditioned by a subthreshold stimulus equal in intensity to 70% of RMT. A subthreshold conditioning stimulus set to 70%RMT was previously shown to result in maximal SICI (Vucic et al. 2009). Stimuli were delivered every 5–10 s (stimulus delivery was limited by the charging capability of the BiStim system) and the computer advanced to the next ISI only when tracking was stable.

*Single*‐*pulse TMS technique* was utilized to determine the MEP amplitude (mV), MEP onset latency (ms) and CMCT (ms). The stimulus response (SR) curve was generated by plotting the peak‐to‐peak MEP amplitude against the absolute stimulus intensity (SI), expressed as a percentage of maximal stimulator output [MSO] and normalized SI expressed as the percentage of MT. The TMS intensity values were as follows; 60%, 80%, 90%, 100%, 110%, 120%, 130%, 140% and 150% of MT. Four responses were recorded at each stimulus intensity and the resultant maximal MEP amplitude, with stimulus intensity set to 150% MT, were expressed as a percentage of the compound muscle action potential (CMAP) response. The CMAP response was generated by stimulating the median nerve at the wrist (see below). The SR slope was calculated from the steepest portion of the SR curve using the following formula:
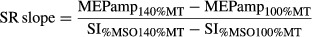


Where MEP_amp140%MT_ and MEP_amp100%MT_ represent MEP amplitude (mV) when normalized stimulus intensity was set to 140% and 100% of MT, respectively. In addition, the SI_%MSO140%MT_ SI_MSO100%MT_ value represents stimulus intensities, as a percentage of the maximal stimulator output (MSO) when the normalized SI is set to 140% and 100% of MT.

The central motor conduction time (CMCT) was derived by utilizing the *F*‐wave method according to the following formula (Mills and Murray 1986). 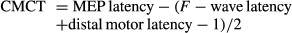


### Peripheral studies

The median nerve was stimulated at the wrist using 5 mm nonpolarizable Ag‐AgCl electrodes (3M Healthcare, St Paul, MN) with the anode positioned ~ 10 centimeters proximal to the cathode over the lateral forearm. Stimulation was computer controlled and converted into current using an isolated linear bipolar constant current simulator (maximal output ± 50 mA; DS5, Digitimer, Welwyn Garden City, UK). The compound muscle action potential response was recorded from the APB with the active (G1) electrode positioned over the motor point and reference (G2) electrode placed over the base of the proximal thumb in a belly‐tendon arrangement. The resultant CMAP amplitude was measured from baseline to negative peak (mV). In addition, the distal motor latency (ms) and minimum *F*‐wave latency (ms) were recorded.

Recordings of the compound muscle action potential (CMAP) and MEP responses were amplified and filtered (3 Hz‐3 kHz) using a Nikolet‐Biomedical EA‐2 amplifier (Cardinal Health Viking Select version 11.1.0, Viasys Healthcare Neurocare Group, Madison, WI) and sampled at 10 kHz using a 16‐bit data acquisition card (National Instruments PCI‐MIO‐16E‐4). Data acquisition and stimulation delivery were controlled by QTRACS software. Temperature was monitored with a purpose built thermometer at the stimulation site.

### Statistical analysis

SICI was measured as the increase in the test stimulus intensity required to evoke the target MEP. Inhibition was calculated off‐line as follows (Vucic et al. 2006): 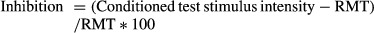


Facilitation was measured as the decrease in the conditioned test stimulus intensity required to evoke a target MEP.

Each data point was weighted [by the QTRACS software] such that any measures recorded outside the threshold target window, defined as values within 20% of the tracking target of 0.2 mV [peak‐to‐peak], contributed least to the data analysis. All results were expressed as the mean ± standard error of the mean. Paired samples *t*‐test was used for assessing differences between two groups. Analysis of variance, with a Bonferroni correction, was used for multiple comparisons. Pearson's correlation coefficient was utilized to assess association between variables. A probability (*P*) value of <0.05 was considered statistically significant.

## Results

Prior to undertaking cortical excitability studies, peripheral nerve function was assessed. There was no significant difference in the CMAP amplitude (CMAP amplitude _REST_ 8.4 ± 0.7 mV; CMAP amplitude _PRECISION GRIP_ 8.4 ± 0.6mV; CMAP amplitude _POWER GRIP_ 8.3 ± 0.8 mV, *P* = 0.44) and distal motor latencies (DML_REST_ 4.0 ± 0.1ms; DML _PRECISION GRIP_ 4.1 ± 0.1 ms; DML _POWER GRIP_ 4.2 ± 0.1 ms, *P* = 0.21) between the three hand tasks, and the values were within the normal range (Vucic et al. 2006). In addition, the minimum *F*‐wave latencies were also comparable between the three hand tasks (*F*‐wave latency _REST_ 29.5 ± 0.6 ms; *F*‐wave latency _PRECISION GRIP_ 29.6 ± 0.6 ms; *F*‐wave latency _POWER GRIP_ 29.6 ± 0.6 ms) and all were within the previously established control ranges (Vucic et al. 2006).

### MEP amplitude

**Figure 1.**
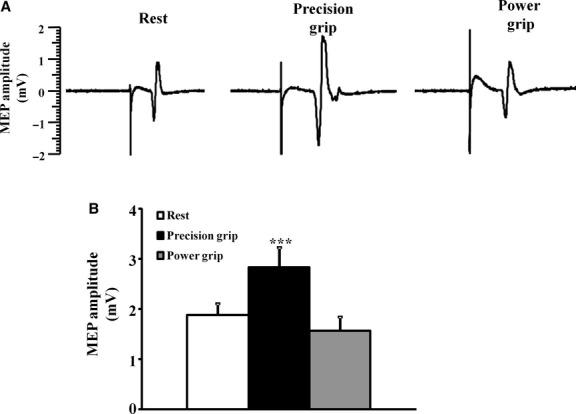
(A) The motor‐evoked potential (MEP) amplitude was significantly increased during precision grip as illustrated in one subject. (B) The group mean MEP amplitude, generated with stimulus intensity set to 150% of motor threshold, was significantly increased during the precision grip. ***P < 0.001

**Figure 2.**
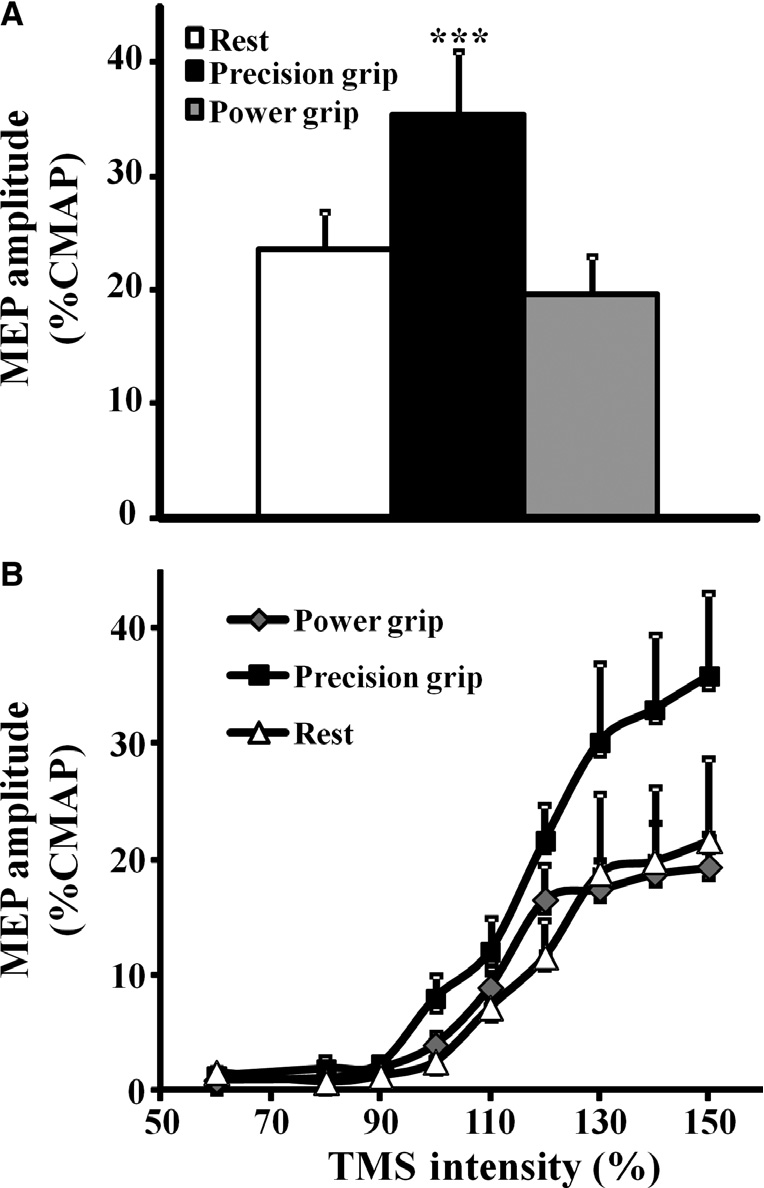
(A) The group mean motor‐evoked potential (MEP) amplitude, expressed as a percentage of the compound muscle action potential (CMAP) response and generated by stimulus intensity set to 150% of motor threshold, was significantly greater during the execution of the precision grip. (B) The magnetic stimulus response curve was also significantly increased during the performance of the precision grip. ***P < 0.001.

A complete sequence of recordings was obtained from all subjects. The changes in MEP amplitude with task‐dependent positioning of the hand are depicted from one illustrative subject (Fig. 1A). In most of the subjects (80%), the MEP amplitude was increased during the precision grip. Importantly, the mean maximal MEP amplitude, with stimulus intensity set to 150% MT, was significantly increased during the precision grip when compared to the hand at rest and in the power grip position (MEP amplitude _REST_ 1.9 ± 0.2 mV; MEP amplitude _PRECISION GRIP_ 2.8 ± 0.4 mV; MEP amplitude _POWER GRIP_ 1.6 ± 0.3 mV, *P* < 0.001, Fig. 1B). Of further relevance, the mean maximal MEP amplitude, expressed as a percentage of the CMAP response and generated with stimulus intensity set to 150% MT, was significantly increased during the precision grip (MEP amplitude _REST_ 23.6 ± 3.3%; MEP amplitude _PRECISION GRIP_ 35.2 ± 5.6%; MEP amplitude _POWER GRIP_ 19.6 ± 3.4%, *F* = 2.4, *P* < 0.001, Fig. 2A).

**Figure 3.**
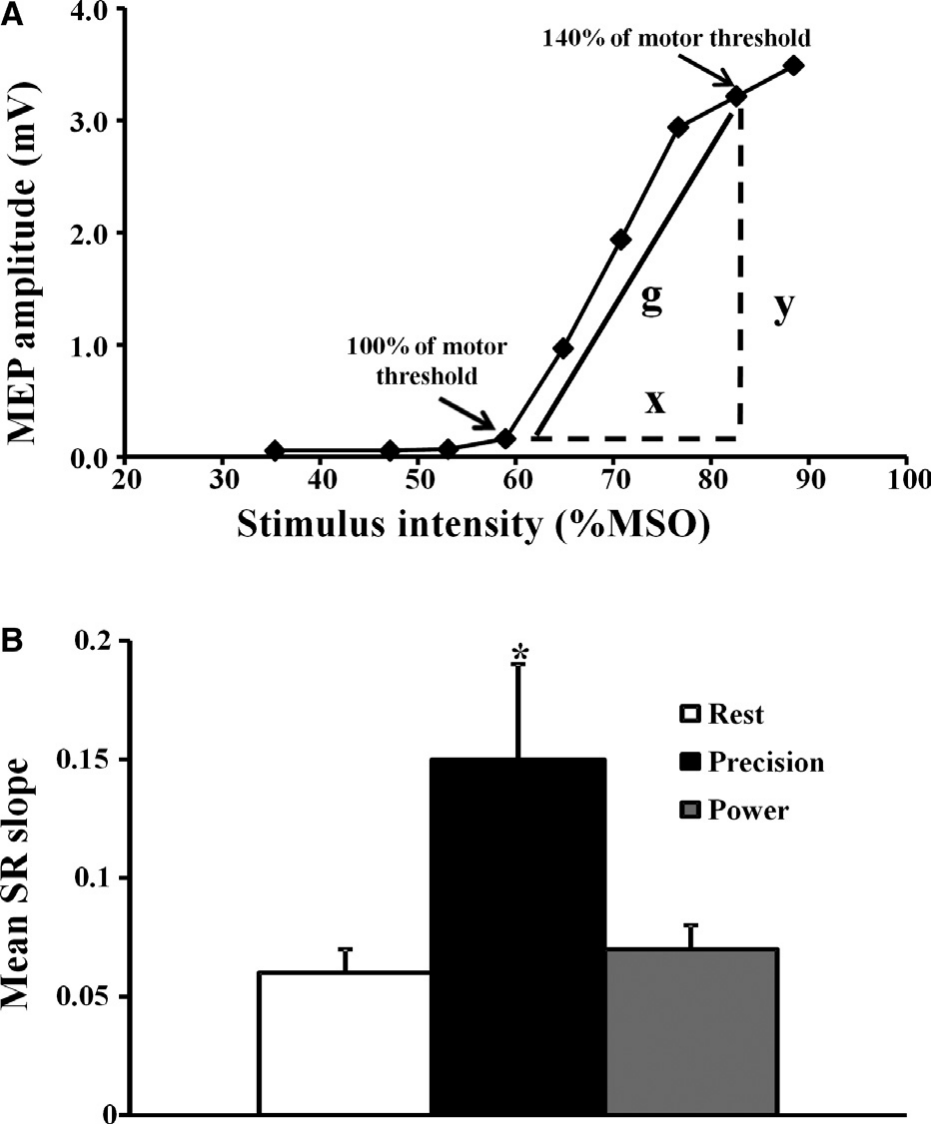
(A) The slope (g) of the stimulus response curve (SR), depicted in one representative subject, was calculated by dividing the motor‐evoked potential (MEP) amplitude (y) by the stimulus intensity (x), expressed as a percentage of maximal stimulator output (%MSO). (B) The group mean SR slope was significantly increased during the precision grip. *P < 0.05.

The magnetic stimulus response curve was significantly shifted to the left and upwards during the execution of the precision grip (Fig. 2B). Of further relevance, the group mean SR slope (see Method and Fig. 3A for calculation) was significantly increased during the precision grip when compared to the hand at rest and during performance of the power grip (SLOPE_REST_ 0.06 ± 0.01; SLOPE_PRCISION GRIP_ 0.15 ± 0.04; SLOPE _POWER GRIP_ 0.07 ± 0.01, *P* < 0.05, Fig. 3B).

### Short interval intracortical inhibition and intracortical facilitation

**Figure 4.**
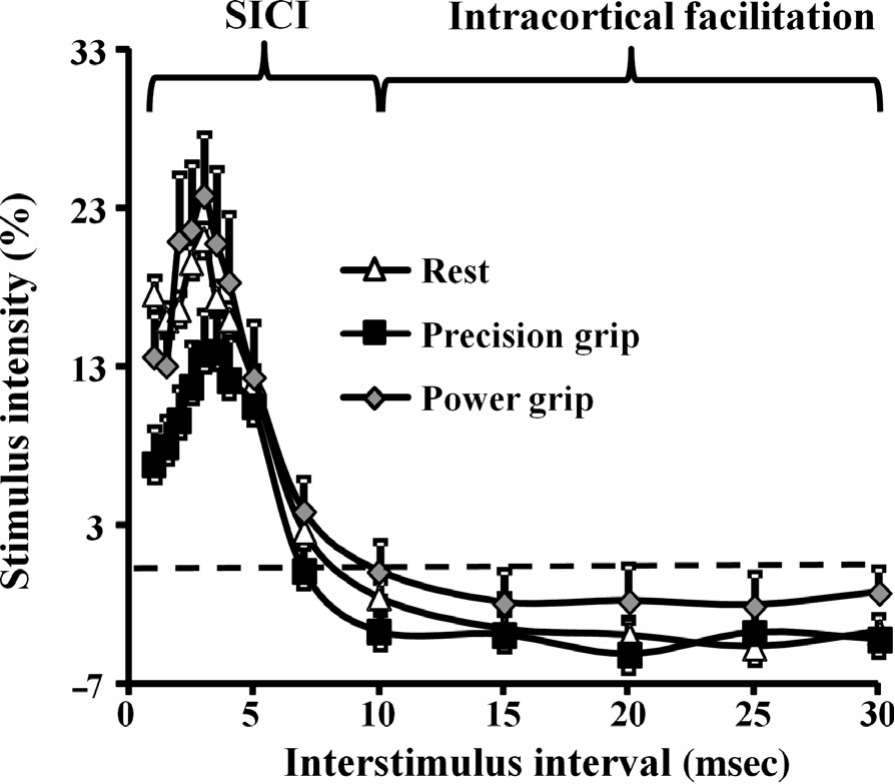
(A) Short interval intracortical inhibition (SICI), as reflected by greater conditioning stimulus intensity required to produce and maintain the target response of 0.2 mV (see Methods), was significantly reduced during the precision grip. In contrast, there was no significant change in intracortical facilitation.

**Figure 5.**
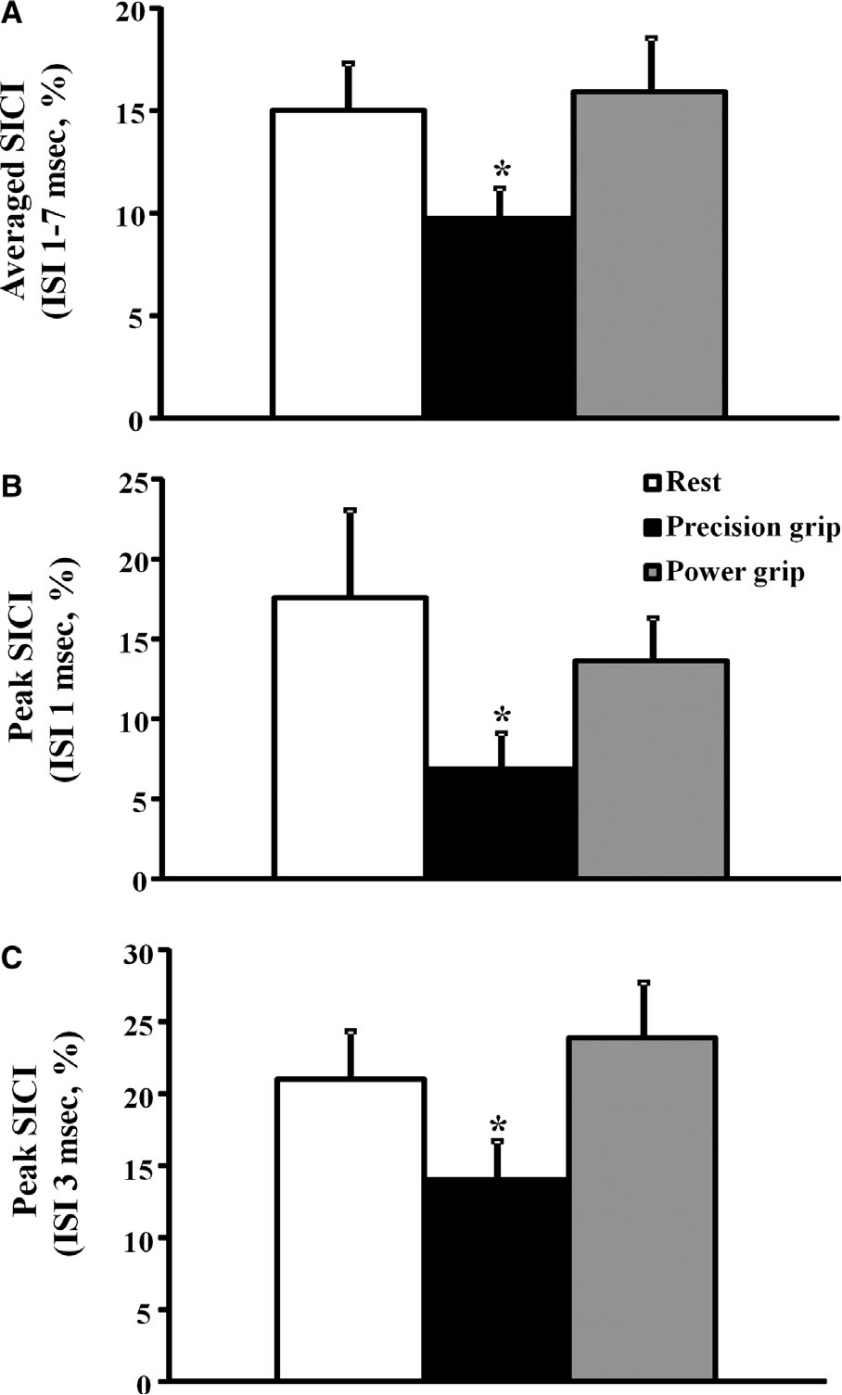
(A) The averaged short interval intracortical inhibition (SICI), between interstimulus interval (ISI) of 1–7 ms, was significantly reduced during the precision grip. Peak SICI at (B) ISI 1 ms and (C) ISI 3 ms were significantly reduced during the precision grip. *P < 0.05.

A paired‐pulse threshold tracking paradigm was utilized to assess the degree of intracortical inhibition. *Short interval intracortical inhibition*, defined as the conditioned stimulus intensity required to produce and maintain the target MEP response of 0.2 mV, was significantly reduced during precision grip (Fig. 4). The averaged SICI, between ISIs 1–7 ms, was significantly reduced during precision grip (SICI _AVERAGED 1–7 ms REST_ 15.0 ± 2.3%; SICI _AVERAGED 1–7 ms PRECISION GRIP_ 9.7 ± 1.5%; SICI _AVERAGED 1–7 ms POWER GRIP_ 15.9 ± 2.7%, *F* = 2.6, *P* < 0.05, Fig. 5A). Importantly, there was a significant correlation between changes in SICI and the slope of the S/R gradient (*R* = −0.38, *P* < 0.05), suggesting the importance of cortical disinhibition in increasing cortical output during maintenance of the precision grip.

Previously, two physiologically distinct phases of SICI have been reported peaking at ISI of 1 and 3 ms. Importantly, peak SICI at ISI 1 ms (SICI _REST_ 17.6 ± 5.5%; SICI _PRECISION GRIP_ 6.8 ± 2.3%; SICI _POWER GRIP_ 13.6 ± 2.7%, *P* < 0.05, Fig. 5B) and at ISI 3 ms (SICI _REST_ 21.0 ± 3.3%; SICI _PRECISION GRIP_ 13.9 ± 2.8%; SICI _POWER GRIP_ 23.8 ± 3.9%, *P* < 0.05, Fig. 5C) were significantly reduced during the precision grip.

Following SICI, a period of intracortical facilitation may develop between interstimulus intervals of 10–30 ms. There was no significant increase of intracortical facilitation with the precision grip (Fig. 3, *P* = 0.12).

### Motor threshold and central motor conduction time

Motor threshold was defined as the stimulus intensity required to produce and maintain the target MEP response of 0.2 mV (see Methods). Surprisingly, there were no significant differences in resting motor threshold between the three hand positions (RMT_REST_ 54.8 ± 2.5%; RMT_PRECISION GRIP_ 50.3 ± 1.7%; RMT_POWER GRIP_ 52.6 ± 2.5%, *P* = 0.14). Of further relevance, there was no significant differences in the central motor conduction time during task‐dependent positioning of the hand (CMCT _REST_ 5.1 ± 0.4 ms; CMCT _PRECISION GRIP_ 4.8 ± 0.2 ms; CMCT_POWER GRIP_ 5.2 ± 0.4 ms, *P* = 0.32).

## Discussion

Findings from the present study have established significant changes in motor cortex excitability during maintenance of the precision grip. Specifically, the motor‐evoked potential (MEP) amplitude and steepness of the input/output plot was significantly increased while short interval intracortical inhibition was reduced during maintenance of the precision grip. In addition, the two previously established phases of SICI were also significantly reduced during the precision grip. These findings suggest that an increase in motor cortex excitability, as indicated by a reduction of SICI and enhanced corticomotoneuronal output, is associated with performance of the precision grip, but not power grip. Taken together, the findings from the present study establish the importance of motor cortical networks in the precision grip, with evidence of motor cortical disinhibition and enhanced cortical output, thereby suggesting that maintenance of the precision grip may be synergistically controlled by a descending corticomotoneuronal drive. The neural mechanisms underlying these task‐dependent changes in cortical excitability will form the basis of the discussion.

### Neural processes meditating task‐dependent motor cortical excitability changes

The MEP amplitude may be a biomarker of the descending corticomotoneuronal drive onto the alpha motor neurons, as well as other noncorticomotoneuronal inputs, which appears to be finely balanced for the mechanical forces required in execution of specific hand tasks (Chen et al. 2008; Quinlan 2011). Of further relevance, the steepness of the magnetic stimulus intensity curve reflects the recruitment gain of the specific corticospinal pathways (Devanne et al. 1997). The findings in the present study of increased MEP amplitude and steepness of the SR curve during precision grip is in keeping with previous studies (Flament et al. 1993; Nakamura et al. 1997; Huesler et al. 1998; Hasegawa et al. 2001; Kouchtir‐Devanne et al. 2012), and suggests that a greater corticomotoneuronal drive to spinal motor neurons, with potential functional coupling, is important in controlling thumb and digit muscles in complex hand tasks. Importantly, these task‐dependent changes in cortical excitability seem to be specific for precision grip, not being evident during power grip, underscoring the specificity of functional coupling of descending corticospinal pathways in controlling thumb and finger movements during the precision grip.

A potential mechanism underlying the enhanced corticomotoneuronal drive during the execution of a precision grip, and thereby functional coupling of circuits controlling thumb and digit force, may relate to enhanced cortical excitability. Specifically, it has been well established that short‐interval intracortical inhibition reflects a balance between the stronger motor cortical inhibitory tone, mediated by cortical inhibitory interneurons acting via GABA_A_ receptors, and weaker excitatory intraneuronal circuits (Chen et al. 2008; Vucic et al. 2013). Underscoring the notion of a cortical origin of SICI are recordings of descending corticospinal volleys through cervically placed epidural electrodes whereby SICI is associated with a reduction in the number and amplitude of late I‐waves, namely I2 and I3, with I‐wave suppression remaining up to an ISI of 20 ms, the typical time course of inhibitory postsynaptic potential mediated through GABA_A_ receptors (Nakamura et al. 1997; Di Lazzaro et al. 1998, 2000; Hanajima et al. 1998).

The finding of a significant reduction in SICI during precision grip, which was not evident during the power grip, suggests that downregulation of inhibitory intracortical network function may be important in the execution of specific hand tasks. Previous studies have reported similar findings, with SICI reduction occurring during the precision grip but not finger abduction (Kouchtir‐Devanne et al. 2012). SICI was also reduced during upper limb pointing tasks requiring co‐ordination of a multiple muscle groups (Devanne et al. 2002). Importantly, inhibition of motor cortical GABAergic neurotransmission in nonhuman primates degrades the independence of finger movements, interferes with task‐specificity of corticomotoneurons and leads to coactivation of agonist and antagonist muscles during movements (Matsumura et al. 1991, 1992; Schieber and Poliakov 1998). Taken together, the present study underscores the importance of SICI in controlling fine motor hand tasks, implying that functional coupling of motor cortical areas representing task‐specific muscles is potentially mediated by disinhibition and excitation of intracortical circuits.

Separately, the present study may shed further light into the neural processes underlying SICI. While it is generally accepted the second phase peaks of SICI, is mediated by synaptic processes, there remains debate as to the initial phase of SICI at ISI 1 ms (Kujirai et al. 1993; Ziemann et al. 1996a,b; Fisher et al. 2002; Roshan et al. 2003; Vucic et al. 2006; Muller‐Dahlhaus et al. 2008). While some have argued that the initial phase of SICI may reflect axonal refractoriness of cortical interneurons (Fisher et al. 2002), our group and others have proposed that synaptic processes were responsible for the SICI at ISI 1 ms (Roshan et al. 2003; Vucic et al. 2006, 2009, 2011). The findings that precision grip resulted in a homogenous reduction of SICI, including the reduction of initial [ISI 1 ms] and later phases [ISI 3 ms], while power grip did not lead to appreciable changes in SICI, provides additional evidence for the notion that synaptic processes appear to be the major mechanism in SICI generation. It remains likely though, that different inhibitory circuits were responsible for these different phases of SICI.

It could be argued that these task‐dependent changes in cortical excitability represent differences in motor thresholds, secondary to EMG activity. Specifically, activation of the target muscle may increase the MEP amplitude, shorten the MEP latency, reduce motor thresholds and SICI (Fisher et al. 2002; Chen et al. 2008). A significant contribution of EMG activity to the current findings seems unlikely, given a comparable level of muscle activation between precision and power grip, and the fact that the MEP latencies and MTs were not significantly different. In addition, it could also be argued that differences in hand positioning between the resting positions (supinated) and precision grip (semi‐pronated) may contribute to the observed findings. Specifically, proprioceptive feedback from the hand is likely to be different between the precision and power grips, and given that transcortical feedback effects are considered to be specific (Scott 2012), a contribution of subtle differences in limb positioning to the TMS findings could not be discounted.

### Clinical implications

The development of task‐dependent changes in motor cortical excitability, with implications that functional coupling of descending corticospinal pathways is important in controlling thumb and finger movements during the precision grip, could be of therapeutic importance. Lesion studies in animals and humans have established the importance of descending corticomotoneuronal pathways in the execution of complex hand tasks (Lemon et al. 1996; Lemon 1997, 2008, 2010; Lemon and Griffiths 2005). In addition, reduction of SICI over the contralateral and ipsilateral (unaffected) motor cortex was previously reported in acute stroke and was associated with a greater severity of functional limb impairment (Huynh et al. 2013a,b,c). Of further relevance, normalization of SICI was reported secondary to botulinum toxin therapy and correlated with improvement in limb spasticity, suggesting the importance of modulating maladaptive cortical plasticity as a therapeutic approach (Huynh et al. 2013a,b,c). Consequently, rehabilitative and pharmacological strategies aimed at restoring the cortical processes involved in regulating complex hand tasks, particularly functional coupling of the descending corticomotoneuronal volleys may prove therapeutically useful in humans.

## Conflict of Interest

None declared.
